# Improving design choices in Delphi studies in medicine: the case of an exemplary physician multi-round panel study with 100% response

**DOI:** 10.1186/s12874-020-01029-4

**Published:** 2020-06-15

**Authors:** Rebekka Veugelers, Menno I. Gaakeer, Peter Patka, Robbert Huijsman

**Affiliations:** 1 Emergency Department, Adrz hospital, P.O. Box 15 4460, AA Goes, the Netherlands; 2 Emergency Department, Erasmus MC, P.O. Box 2040, 3000 CA Rotterdam, the Netherlands; 3 Erasmus School of Health Policy & Management, P.O. Box 1738, 3000 DR Rotterdam, the Netherlands

**Keywords:** E-Delphi, Delphi technique, Emergency medicine, Emergency service, Hospital, Response rate, Decision rule, Evidence-based emergency medicine, Epidemiologic research design, Operational standards, Tips and tricks

## Abstract

**Background:**

A proper application of the Delphi technique is essential for obtaining valid research results. Medical researchers regularly use Delphi studies, but reports often lack detailed information on methodology and controlled feedback: in the medical literature, papers focusing on Delphi methodology issues are rare. Since the introduction of electronic surveys, details on response times remain scarce. We aim to bridge a number of gaps by providing a real world example covering methodological choices and response times in detail.

**Methods:**

The objective of our e(lectronic)-Delphi study was to determine minimum standards for emergency departments (EDs) in the Netherlands. We opted for a two-part design with explicit decision rules. Part 1 focused on gathering and defining items; Part 2 addressed the main research question using an online survey tool. A two-person consensus rule was applied throughout: even after consensus on specific items was reached, panellists could reopen the discussion as long as at least two panellists argued similarly. Per round, the number of reminders sent and individual response times were noted. We also recorded the methodological considerations and evaluations made by the research team prior to as well as during the study.

**Results:**

The study was performed in eight rounds and an additional confirmation round. Response rates were 100% in all rounds, resulting in 100% consensus in Part 1 and 96% consensus in Part 2. Our decision rules proved to be stable and easily applicable. Items with negative advice required more rounds before consensus was reached. Response delays were mostly due to late starts, but once panellists started, they nearly always finished the questionnaire on the same day. Reminders often yielded rapid responses. Intra-individual differences in response time were large, but quick responders remained quick.

**Conclusions:**

We advise those considering Delphi study to follow the CREDES guideline, consider a two-part design, invest in personal commitment of the panellists, set clear decision rules, use a consistent lay-out and send out your reminders early. Adopting this overall approach may assist researchers in future Delphi studies and may help to improve the quality of Delphi designs in terms of improved rigor and higher response rates.

## Background

Medical researchers commonly use the Delphi technique for consensus studies. A proper execution of this technique is essential for a study’s validity, but the present medical literature on the topic has so far remained rather vague. In this paper, we discuss several methodological issues and panel response characteristics based on our study amongst an expert panel of Emergency Physicians [1].

The Delphi technique is designed to obtain the most reliable consensus of opinion in a group of experts. It attempts to achieve this by means of a series of questionnaires interspersed with controlled feedback including group statistical responses [2, 3]. Anonymity amongst panellists prevents the occurrence of individual dominance that may result from strong verbalization, track records or professional dominance. It also allows panel members to change their opinion on the basis of arguments presented by the other panel members without publicly admitting that they have done so. These advantages are assumed to increase reliability of consensus [4]. When an online survey tool is applied, the term e-Delphi (electronic) is used.

Since the early nineteen-fifties, the Delphi technique has been widely used in a large number of diverse domains such as the military, business, education, social science and health care [4]. It can be used for a wide range of complex research aims, including forecasting, issue identification, issue prioritization, ranking, policy formation and concept-framework development [5, 6]. However, the method’s versatility is both a strength and a weakness. Practitioners are often willing, and sometimes even eager, to modify Delphi to meet their own decision-making and forecasting needs. In some cases, these modifications are meaningful and contribute to a better understanding of the technique; in other cases, they are random and arbitrary – thus undermining quality and credibility [4].

Reports of medical studies using Delphi often lack detailed information on how Delphi studies are conducted [7, 8]. This may be partly due to medical journal word count limits, but it results in low repeatability and limited insight into external validity. To address this matter, a guideline for reporting (CREDES) was published [9]. Still, most of the existing methodological literature on Delphi will not be found by using common search strategies for medical research in databases such as PubMed and Embase, and medical researchers may therefore easily overlook these. For novel users, however, such information is crucial, because the method’s apparent simplicity is in contrast with the work and the difficulty involved in its proper execution [3]. Time it takes to complete a Delphi study is usually underestimated [10] and respond rates are often disappointingly low [10, 11] Deficient application of the technique will lead to poor validity of the results [3, 5, 12]. In addition, little has so far been published on the way in which panellists respond to the presented rounds, what circumstances contribute to high response rates in a panel or how these can be optimized.

With this paper, we provide insight into the methodological challenges encountered in our Delphi study on ED standards and present the solutions we formulated based on the available data and how these turned out. With our work, we aim to aid others who plan a Delphi Design and to help them improve the quality of their study. In addition, we provide details on response times from the current e-Delphi study.

## Methods

The objective of our e(lectronic)-Delphi study was to determine minimum standards for emergency departments (EDs) in the Netherlands. We based our methodological choices on available literature; we discussed and decided on these within our full research group, and we kept a record of the methodological considerations and evaluations made by the research team before as well as during the study. Per round, the number of reminders sent and individual responses times were noted.

Table 1 presents an overview of items requiring design choices that influence the rigor of a Delphi, based on the CREDES guideline for conducting and reporting [9].

**Table 1:** Designing an e-Delphi using CREDES

● Define purpose and rationale of Delphi	
● Prior information for establishing the knowledge base of the panel	
● Unstructured (classical) or structured (modified) first round	
● Required question type (qualitative or quantitative)	
● Define consensus and non-consensus	
● Clear and transparent guidelines on how to proceed from round to round	
- purpose of rounds	
- what if no consensus is reached after several iterations?	
- do items need to be deleted in next rounds (consensus / rated irrelevant)?	
- do items need to be refined in next rounds (when and how)?	
- number of rounds	
- define what determines the last round	
● Strategy for processing results between survey rounds	
● Development of materials/instruments (platform / lay-out / questions)	
● Pilot materials / instruments	
● Selection of experts	
● Role of research team	
● Strategy to improve response rate	
● Validate final report externally	

### Purpose and rationale of our Delphi

The study that is used as an example in this article achieved a consensus among Emergency Physicians on minimum operational standards for emergency departments (EDs) in the Netherlands [1]. It focused on three domains: ED facilities, diagnostics and the availability of medical specialists. Its background is the rapidly changing emergency health care environment, where crowding, an aging patient population and staff shortages put pressure on the quality and availability of ED care. A clear view on the minimum standards of a hospital-based 24/7 ED is important for policy makers set course toward optimal conditions for delivering adequate care for patients with undifferentiated, urgent or emergent complaints. A second driver for the study can be found in the ED context with highly trained physicians, which may be a challenging field to explore the various methodological aspects to design and perform a rigorous Delphi. Here, we set out to formulate clear and repeatable options for addressing known Delphi methodology issues, and we describe how these played out in our study. We do so in order to contribute to the work of medical Delphi users and assist them with the application of the Delphi technique in practice.

### Prior information for establishing a panel’s knowledge base

Panellists were provided with information on the study’s purpose and rationale, including the definition of ED that was used. Experts remained anonymous and unknown to each other throughout the entire process. One selection criterion for our panellists was a prior possession of the required knowledge base [1], and therefore no additional information on ED operational standards was provided. In this way, the risk of influencing the experts’ judgements was eliminated.

### Unstructured (classical) or structured (modified) first round

In a ‘classical’ Delphi, the first round is unstructured, allowing free scope for experts to elaborate on issues they deem important. Commonly, however, this round is structured to save time and effort for both the monitor team and the panellists [6].

We assumed that interpretation differences amongst panellists could exist with respect to the items involved, and that these could thus affect the discussion of the main research question in a negative manner. To avoid such miscommunication on items, we added Part 1 prior to focusing on the main research goal in Part 2, with both parts consisting of several rounds. In Part 1 we combined the collection of all relevant items from the literature and the panel with reaching consensus among panellists regarding the definition of these items. Additionally, adding a consensus path before introducing the main research question allowed the panel and the monitor team to familiarize themselves with the method and the online survey tool. Such clear definitions will also aid interpretability of the results for others.

We started our study in a semi-structured fashion (Part 1, Round 1): panellists were provided with a list of items and a proposed definition for each of these. Panellists were then asked to do two things. First, they were invited to add all items that could possibly be regarded as an ED facility or a diagnostic option for an ED. To stimulate panel members to submit plain as well as more uncommon items in the first round, we made sure that the provided list included items that panellists would most likely consider to be necessary for every ED in Part 2 (for example, every ED needs a toilet) as well as unnecessary (for example, every ED should have an MRI scanner on the shop floor). Next, the panellists rated and commented on the provided definitions. Once all items had been gathered and consensus on the definitions of all items had been reached, we proceeded with Part 2. Part 2 focused on the main research topic: minimum ED standards in three domains (ED facilities, diagnostics and availability of medical specialists), for which the defined items resulting from Part 1 and a list of all medical specialties were used.

### Required question type (qualitative or quantitative)

In Part 1, panellists were asked dichotomously if they could agree with a proposed definition. Open text fields were used for panellists to explain their choices and to add their opinions on remarks made by their colleagues. Open fields were also used to include any additional items.

In Part 2, the domains ‘ED facilities’ and ‘diagnostic availability’ started dichotomously, but they were converted into multiple-choice options when two or more panel members suggested a similar adjustment or condition. This was added in the next round, for example “agree, only when condition x is satisfied”.

In the third domain (availability of medical specialists), we first aimed at a yes-no level of necessity. Additionally, we wanted more information on the degree to which the panel felt this was necessary. We used a multiple choice approach to limit the time and effort asked from panellists, for example “agree, 24/7 availability” and “agree, 24/7 availability by phone as well as physically available within 30 minutes”. Again, when two panellists added a similar remark, this was added as an answer option. Items that were selected by fewer than two panellists were omitted in the next round, but panellists were offered the opportunity to disagree with the remaining options when they were not convinced by the motivations given by other panellists.

### Define consensus and non-consensus

Consensus was assumed to have been reached when at least 70% agreement was achieved. Our decision rules are presented in Table 2. When consensus was reached, members could ask for an item to be re-discussed, but a motivation was required. Again, a threshold of two similar motivations was needed for an item to be discussed in the next round. We opted for this approach to enhance the validation of the consensus reached. It also provided panellists with the opportunity to individually avoid process loss due to early closure (through reaching agreement on the first solution that nobody strongly objects to with the aim to achieve consensus rather than aiming for the best possible judgment that is agreed upon wholeheartedly by most). The obvious disadvantage in this case is that it required more time and effort on the part of the panellists.

**Table 2:** Defined and applied decision rules

consensus is declared at 70% agreement	
2 similar requests warrant re-introduction of a consensus declared item	
2 similar suggestions for change/addition in answer options result in a matching adjustment (to be discussed again in the next round) regardless of consensus reached	
no consensus after 4 rounds without major change nor suggestions for change is accepted as non-consensus	

### Clear and transparent guidelines on how to proceed from round to round

**Fig. 1:**
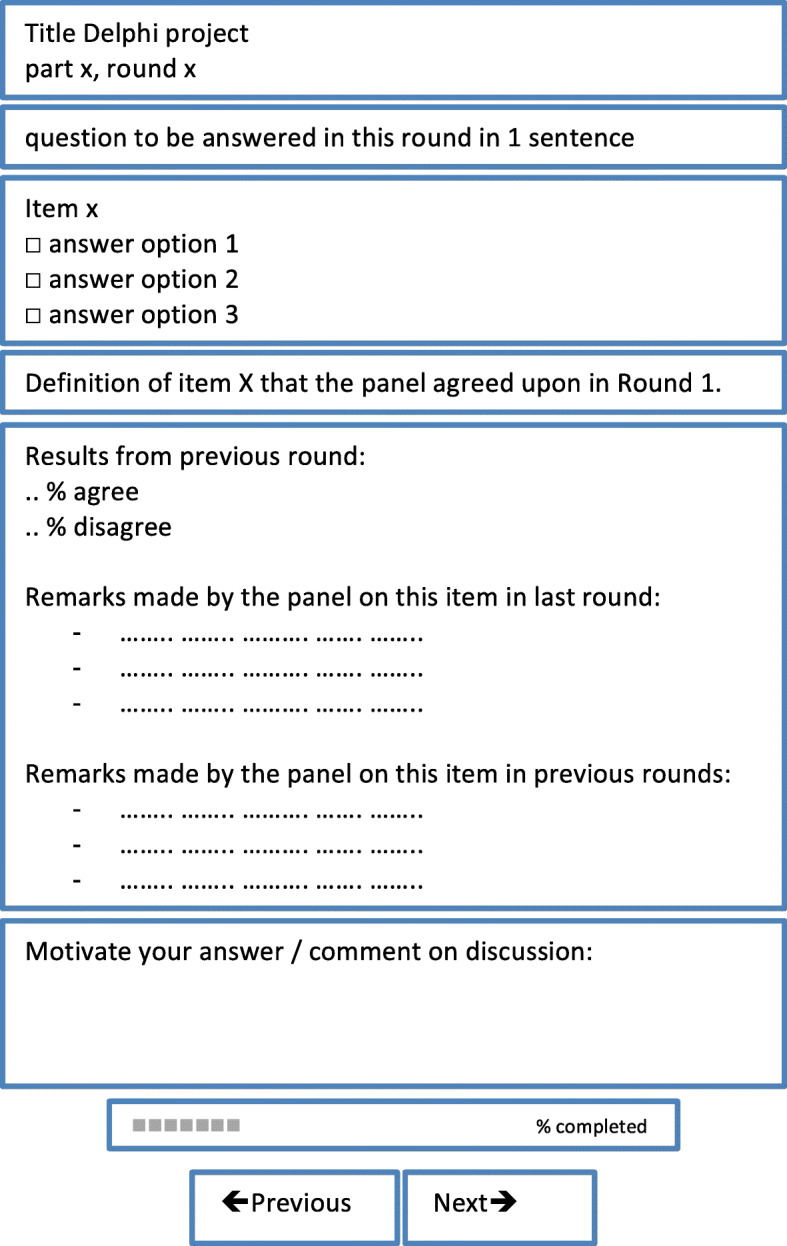
Components of online survey. The design was identical for each page of the survey and consisted of the following components

We decided not to set a fixed number of rounds. Instead, we set endpoints and decision rules indicating when to add changes and when to accept. Classically, the number of iterations seldom exceeds one or two rounds, when most changes in panellists’ responses are expected [6, 13]. However, the possibility to respond to the provided feedback and consensus are essential for improving validity [6]. The downside of multiple rounds could be that panellists (even ‘holdouts’) can become tired of discussing the same item again and tend to lean towards closure and changing their opinion towards the mean if the number of rounds keep increasing [13, 14] or they drop out [11]. We tried to minimize such weariness amongst panellists by making sure the surveys were easy to fill out, with a clear and consistent presentation, in order to minimize the amount of time needed. In the introduction of each round the purpose of each part/round was explained to the panel and decision rules relevant to the specific round were shared. The survey then started with the pages with items on which no consensus had been reached previously (Fig. 1), followed by items on which consensus had been reached in the previous rounds together with all remarks. Only when the panellists did not wish to see an item that had been consented upon earlier return for rediscussing, this item was deleted from the next round.

When a similar suggestion for adjustment of an item was made by at least two panellists, it was changed and the adjusted item was put forward for discussion again in the next round. This rule was also applied when consensus was reached in the same round as suggestions for change were made; we called this reached consensus ‘preliminary consensus’. The item was adjusted and put forward again in the next round (an example is included in the Results section below). When in Part 2, Round 4 no consensus was reached and no suggestions for change were made, nor major shifts in opinion were seen, we accepted non-consensus with respect to these items.

### Strategy for processing results between survey rounds

The online survey tool generated the pooled results per round. Quantitative data were expressed in percentages as statistical group response. Newly generated items were included in the next round. Qualitative data were presented per item; arguments from the last round were presented separately from the arguments from the previous rounds.

Our rule for modification required 2 similar suggestions for adjustment. Two members of the research team (RV and MG) individually judged all comments on similarity. In case of disagreement, the data were discussed in the full research team.

### Development of materials/instruments (platform / lay-out / questions)

An online survey tool was used (SurveyMonkey.com®) with a clear lay-out that was identical in all rounds (Fig. 1). We presented one item per page, including a statistical summary of the former round, and anonymized remarks from prior rounds. Each member of the panel received an individual invitation for each survey. A survey could be closed and continued whenever the panellists wanted this.

### Pilot materials / instruments

Surveys were built per round by one researcher (RV). No formal pilot was performed. The functionality of each round was tested using different mail servers and operating systems and checked by a second researcher. When agreed upon, the survey was sent to the panel and a dummy version was sent to the research team for control purposes.

### Selection of experts

One of the key aspects of a rigorous Delphi study is the selection of experts for the panel, as this is crucial for the validity of the resulting consensus. Therefore, much thought should be given to assembling a representative panel. Steps in this selection process include identifying the classes of experts, completing these with names, contacting individuals and letting them nominate others for inclusion, ranking individuals on the basis of qualifications, inviting individuals and finally asking for their commitment [5]. Gaining commitment from high-profile and busy panellists depends on the way in which this process evolves. Incentives to participate could include being invited to join a selective group and the opportunity to learn from the consensus process [5]. We did not pay our experts, nor did we give them presents of any kind. The ideal number of panellists for a Delphi is not set in stone, but a recommended number is 10 to 18 for ensuring the development of productive group dynamics and for arriving at consensus among experts [9, 15].

We recruited our panel among Emergency Physicians (EPs) to guarantee a broad but direct clinical view on the topic. We added the following requirement: 15 to 25 members had to be included from all over the country (i.e. one to two members from each of the country’s 11 predefined regions of acute care), and there had to be a balance between working in academic, semi-academic and rural hospitals. We aimed for EPs with demonstrated competence in management or education (Table 3), added names to our list and called the highest-ranking EP in each region. Calls were made by MG, a Dutch EP well known by most EPs as the former Chairman of the Dutch Society of EPs. EPs were informed about the aim of the study, the methodology and the importance of anonymity, and we emphasized the amount of the time that they needed to invest. When other EPs were nominated, we ranked them on our list. Verbal and written informed consent was obtained from all panellists.

**Table 3:** Composition of expert panel

Number of panel members	20
Registered Emergency Physician	20
Working clinically in a 24/7 hospital-based ED	20
Experience as ED manager	10
Experience in sector or hospital management	11
Experience with ED design within last 5 years	14
Experience as educator in ED specialty training	16
At least 3 years of working experience as registered EP	19
LNAZ region	
Groningen	2
Zwolle	1
Enschede	2
Nijmegen	2
Maastricht	2
Tilburg	1
Rotterdam	2
Utrecht	2
Leiden	2
Amsterdam VUMC	2
Amsterdam AMC	2
Hospital type	
academic	6
semi-academic	8
rural	6

### Role of research team

The research team designed the study and set the decision rules, in line with the available literature. They applied these rules and closely monitored and evaluated the methodological aspects of the study and managed the overall study process. The summary statistics and panellists’ input were interpreted and discussed by the full research team. Minutes of these meetings were kept in a log. Members were careful not to influence panellists’ opinions, and none of the members had a conflict of interest.

### Strategy to improve response rate

Activities to improve response rates started as early as the inclusion process for the panel’s members, when each panellist had personal contact with the main researcher (MG). Response rates can be enhanced by a ‘personal touch’ together with a clear explanation the study process and awareness of the importance of commitment to the study for the validity of the results [11].

After inclusion, panellists received an e-mail with study details and additional message in the week leading up to each round. The link to the survey was sent by e-mail and a text message was sent by phone. When the research team felt it was necessary, panellists received a reminder. This was done on the basis of prior response times of the individual panellists, expected duration of completion and national holidays. Panellists were prompted by one researcher (MG).

### Final draft

According to CREDES, results from a Delphi study should be reviewed by an external board before implementation. However, since emergency care is a multidisciplinary field and support needs to be found amongst the various emergency care stakeholders, our study consensus was not set up for direct implementation [1], since emergency care is a multidisciplinary field and support amongst the various emergency care stakeholders; it was the starting point for a further discussion about the development of standards for EDs.

## Results

### Panel participation

The expert panel consisted of 20 EPs. Their experience and backgrounds are shown in Table 3. Only one EP nominated a more experienced colleague, who agreed to participate instead. Per round, we sent a maximum of two e-mails with the link to the survey, up to four reminders, and we made a maximum of two calls. This led to a 100% response rate without any dropouts.

### Item throughput

**Fig. 2:**
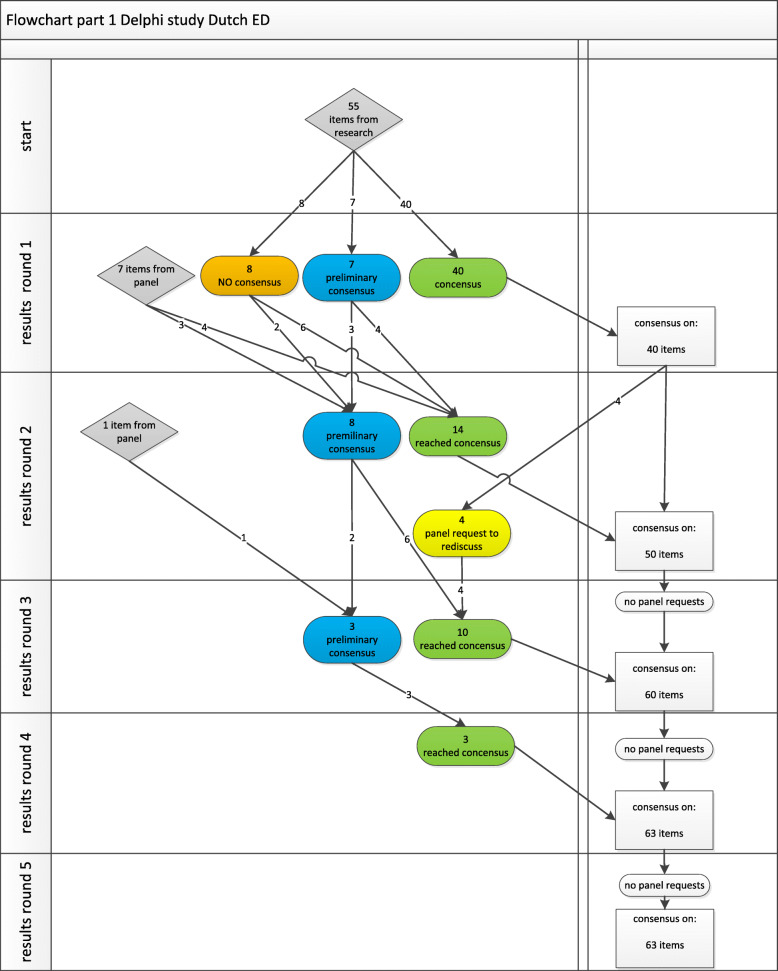
Flowchart for Part 1: gathering and defining items. Preliminary consensus = consensus (agreement 70% or above) but retained based on other decision rule

**Fig. 3:**
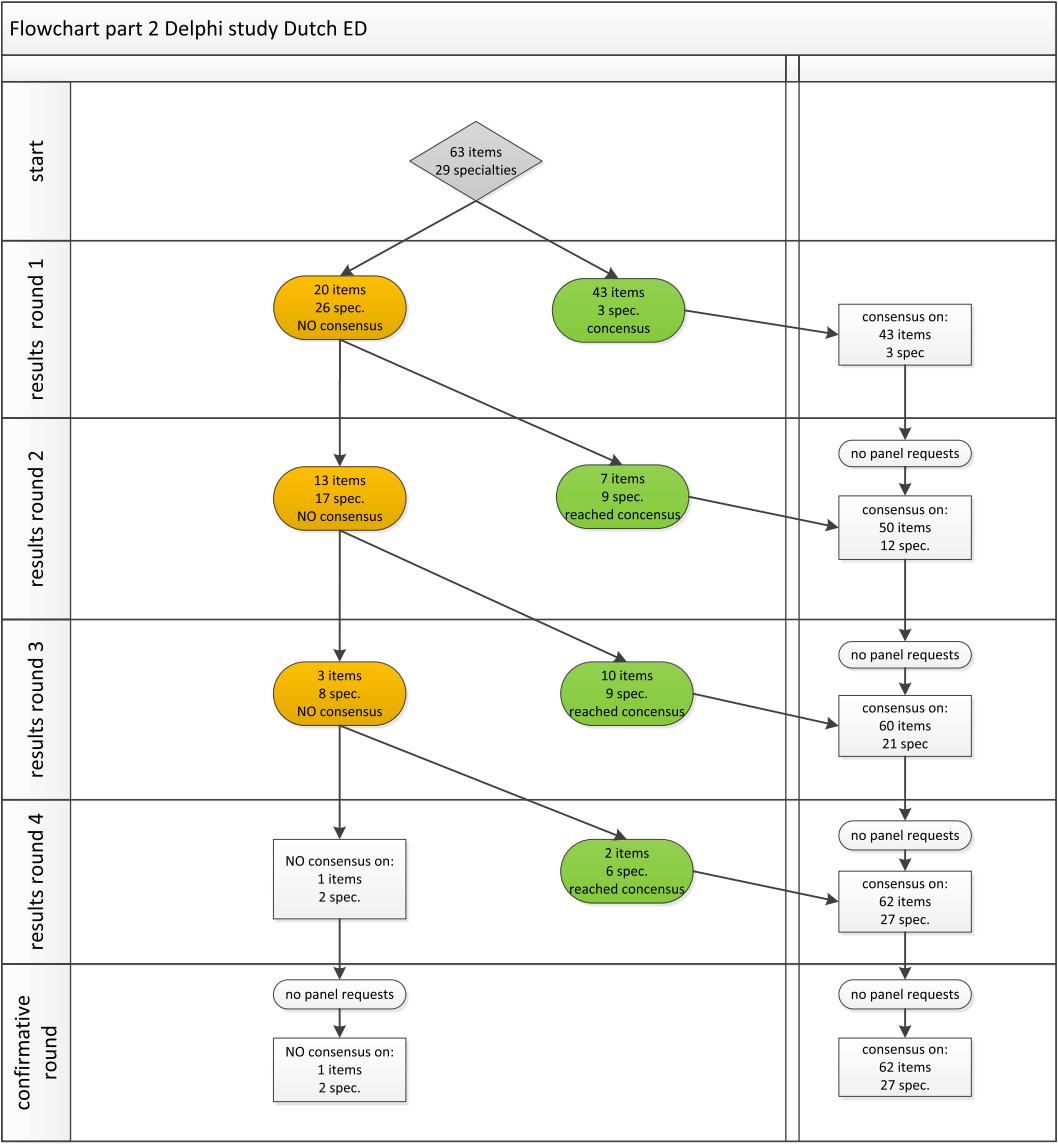
Flowchart for Part 2. the main research topic: minimum ED standards in three domains (ED facilities, diagnostics and medical specialist availability)

Part 1 started with 55 items. After four rounds, no items were requested back for re-discussion, and this left us with 63 items and consensus on definitions for all of these (Fig. 2). In Part 2, we started with these 63 items and added 29 medical specialties. After four rounds, this resulted in consensus on 62 (98%) items and 27 (93%) specialties (Fig. 3), and no items were requested back.

### Percentages agreement

The first round in Part 1 resulted in an average agreement of 85% per item, varying from 55 to 100% for different items. For items in which consensus was reached in the first round, average agreement was 89%. The average agreement percentage in the non-consensus group was 63%. If an item needed several rounds to reach consensus, the ultimate average agreement percentage for this item was higher: 98% vs 89%.

We clearly experienced the added value of the decision rule of continuing when two panellists submit similar suggestions for adjustment, irrespective of the agreement percentage: definition agreement for CBRN room (room for treating patient with Chemical, Biological, Radiological and Nuclear contamination) was 79% in Round 2, which was enough agreement to call this a consensus. However, because more than one panellist had made a similar remark for adjustment, we continued the discussion. The results that followed led us to split the definition into two separate items (CBRN room minimum option and CBRN room maximum option), and in the next round 90% consensus was reached for both items. The process was then repeated again, based on the same decision rule, which resulted in 100 and 95% consensus, respectively.

In the first round of Part 2, we found an average agreement of 79% (50–100%) with respect to facilities and 77% (50–100%) with respect to diagnostics. For those items (facilities and diagnostics) in which consensus was reached in the first round, average agreement was 87%. Average agreement on items that did not reach consensus in Round 1 was 59%.

In Part 2, a third domain (availability of medical specialists) was added. On the basis of a yes/no scale, this resulted in agreement for 90% of the 30 medical specialties in the first round; this remained 90%. On the scale used for the degree of this availability, there was little agreement in the first round (only for 10% of specialties), but in the final round consensus was reached for 90% of specialties. A striking change was seen for plastic surgeon necessity: in Round 1, 15% of our panellists supported the idea that a plastic surgeon was not necessary in every ED (= consensus on ‘necessary’), but this changed to 95% (= consensus on ‘not necessary’) when, based on panellists’ input, an option was added for the condition that regional availability should be guaranteed instead.

### Agreement with regard to regarded necessity

**Fig. 4:**
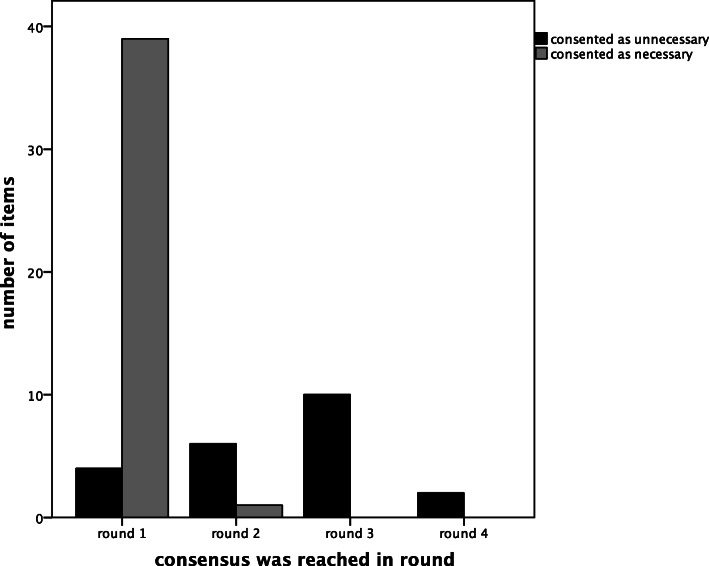
Deemed necessity and influence on number of rounds to reach consensus

For items (facilities and diagnostics) that were deemed nessesary, consensus was reached in the first round for 97% of the items concerned (3% in Round 2). When items were considered unnecessary, consensus was reached in the first round for 18% of the items (27% in Round 2, 45% in Round 3, 5% in Round 4, 5% in Round 5). Items that resulted in consensus on necessity mostly took one round, whereas items that resulted in consensus on non-necessity needed an average 2.45 rounds (Fig. 4). Of the 22 items that were considered unnecessary, there were 13 items for which the panel set the condition that (part of) their functionalities should be available in another way before consensus was reached.

### Panel participation and response

**Fig. 5:**
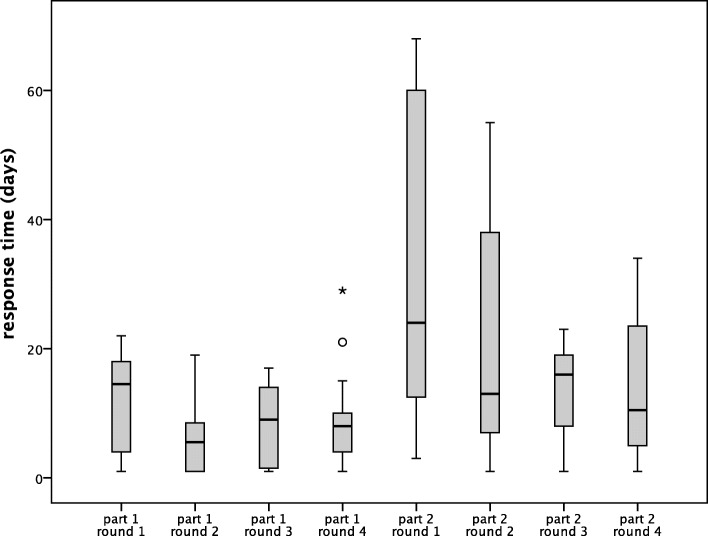
Median time to complete survey per round. The vertical axis displays the response time in days; the horizontal axis lists the individual rounds. The figure represents the median, the interquartile range and two outliers in Part 1 Round 4

We found large differences in response time between panellists and between rounds (Fig. 5). Response time consists of the time needed to fill in a questionnaire as well as waiting time until the panellist opens the survey. In our study, once panellists started filling in the questionnaire, they nearly always finished the questionnaire on the same day (on average 80%). We did not keep data on how often and for how long the survey was accessed.

**Fig. 6:**
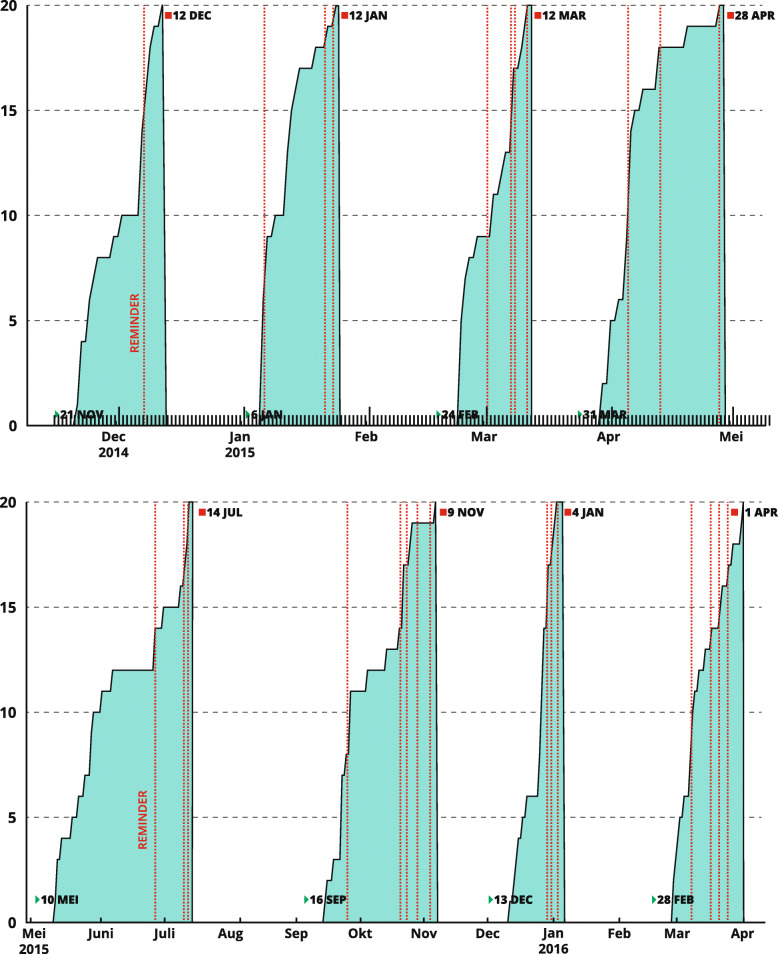
Response times and reminders. The horizontal axis presents the date; the vertical axis presents the cumulative returned surveys. The dotted lines represent one or more reminders that were sent

**Fig. 7:**
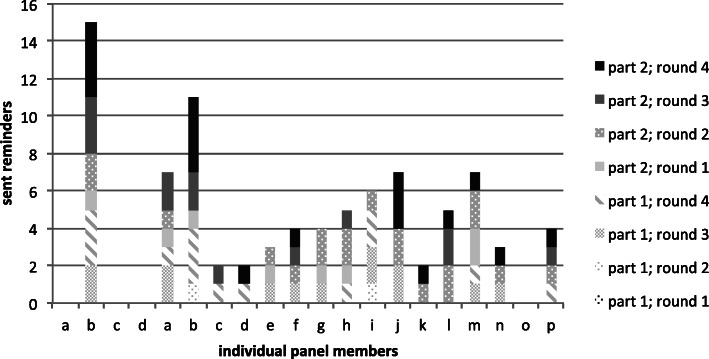
Number of reminders sent to individual panellists in total and per round

In all rounds, panellists were prompted to respond. The timing and method of sending reminders (see Fig. 6) was not standardized. Timing depended upon the expected time needed to respond perceived by the research group. This was based on i.e. the panellist’s previous responses, the expected time investment and national holidays. Three panel members did not need any prompts, and one member was prompted 15 times over the eight content rounds (Fig. 7). In total, these Delphi rounds took 17 months to complete.

## Discussion

The quality of a Delphi study strongly depends on its design and the quality of its execution. In 2017, guidelines were published on the execution and reporting of such studies (CREDES). Since then, thousands Delphi studies have been published, but the use of CREDES has not been generally adopted. The Equator network used a 4 item quality score for reporting [16] all of which are also included in CREDES. Nevertheless, is a remaining clear call remains for a Delphi standardization [17] and researchers continue to study the method itself [16, 18–20] with the aim to improve Delphi standardization and quality. However, little has so far been published on practical design choices and how these ultimately play out. This paper tries to fill some of these gaps.

In the first part of our two-part design, we not only collected possible items but also established a common language. We could not find previous studies that did this in a similar way. Defining items took several rounds confirming our assumption that individual items can be interpreted differently by different panellists. After Part 1 had been successfully completed, there appeared to be little miscommunication in the panel responses in Part 2. Another advantage of our two-part structure was that both the panel and the research group became acquainted with the method before focusing on the main research question. A disadvantage may be the time investment needed for extra rounds, but in view of our 100% response rate this does not have to be a problem. Another downside was that panellists were so eager to start answering the main research question that they found it hard to limit themselves to the definitions considered in Part 1. Keeping a steady focus also remained difficult in Part 2, as panellists tended to dwell on the items in other situations. For example, panellists could present arguments specifically related to EDs in cardiac intervention centers, although our study explicitly focused on the minimum requirements for 24/7 EDs only, regardless of size or type.

There are no strict guidelines on the correct number of rounds. The number of rounds is either set in advance or rounds continue until consensus is reached; no further changes take place or return rates diminish [10]. Having a total number of four rounds in Part 1 and four rounds in Part 2 did not influence the response rate negatively. A low number of rounds is generally thought to increase completion rates, but clear evidence is lacking. We have shown, as was described previously, that response rates can stay high (even as high as 100%) with a high number of rounds. This was previously attributed to a highly motivated panel and multiple reminders [17, 20]. To avoid shifting opinions due to study fatigue, it must be made clear to panellists that they do not need to strive for conformity, and that the study will end when no further changes in opinion are presented. We decided to apply clear and strict decision rules to reduce the risk of bias due to subjectivity or inappropriate influence from the research team. Our decision rules worked out well and proved to be applicable without disagreement on their interpretation, and no adjustments or violations were needed. Our approach structured the interpretation of the panellists’ input and the response of the research team. We added a ninth and final confirmative round to offer panellists the option to ask for items to be returned in order to be discussed again. Although no items were asked back, this made sense for two reasons. Firstly, it is in line with our decision rules, which therefore makes our work methodologically sound. Secondly, this could resolve the issue recently raised that true consensus is not merely a majority of vote, considering that some could find an outcome unacceptable [21].

Questions about the first two domains were presented with yes/no answers and free text to elaborate on the rationale behind panellists’ choices and to add remarks on results from previous rounds. This proved to be effective. For the third domain (availability of medical specialists), we used a structured approach. We wanted yes/no answers as well as opinions on the way in which options should be put into place. We selected multiple options based on common practice (yes, 24/7; yes, on call < 30 min, etc.). In hindsight, we would approach this differently next time. A better option would be to ask yes/no questions and to add a compulsory text box for panellists to indicate what they believe the minimum availability option would be. Such an approach could stimulate out-of-the-box suggestions and possibly create support for such an option in the next round.

Building an online Delphi study requires suitable software and effective lay-out choices. A specific Delphi software programme that provides structure and supports feedback reporting could improve such a study [21]. In the absence of major players in this specific field, we selected a well-known and commonly applied survey tool. We used clear and identical formats in each round. Each round started with a short introduction detailing the necessary background information and the study’s progress, and each page stated the objective of the study. This is in line with the given advice to be clear about the objective [19], and repeatedly specifying the objective keeps panellists focused on the goal [17, 20]. After the second round in Part 1 of our study, we made all essential questions compulsory and kept filling in the text boxes optional. In hindsight, as mentioned above, this should have been done from the start. Finally, we retained the option to stop and restart at any time, and we also retained the option to go back to the survey at a later moment to make changes or additions for as long as the round was open. This remains a logical choice.

Considerations based on social influencing principles, for example in view of the need for blinding, are of significant importance in the Delphi technique. Generally reported reasons for the blinding of panellists are the following: avoiding group pressure, avoiding situations in which people gear their opinions towards those expressed by the most dominant or most highly respected panellist and ensuring that panellists feel free to change their views. In addition, similar choices based on social principles can help to improve response rates. This was previously suggested by McKenna [11, 22] as the ‘personal touch’. In our study the main researcher recruited participants personally by phone. This added not only the effects of liking [23] (knowing each other and having similar goals), but also authority [23] (being a well-known and highly respected colleague) as well as reciprocity [23] (service as provided in the past by being chairman). Setting a similar goal during the recruitment process (a consensus standard as a means towards improving the ED landscape) strengthened the principle of liking. This type of information and personal contact during the recruitment process most likely set the basis for the project’s success.

No single best strategy is known for sending out reminders, but persistence is felt to be important [10]. We decided to individualize reminders. Considering our 100% response rate, we may conclude that this was an effective strategy. It has been recently shown that most panellists do not disapprove receiving reminders [20]. However, there is still room for improvement; for example, sending reminders at earlier moments might have shortened response delays in the first round of Part 2. That said, response delays in our study were very rarely due to the time that was needed to complete a survey, but almost always due to delays in opening and starting the survey. Once a panellist had started the survey, the questionnaire was almost always finished in 1 day. Individualizing reminders seemed justified since interpersonal differences proved to be large, while intrapersonal differences were limited. For example, one panellist needed more than one reminder for all but the first survey round, while some needed none, and others needed one reminder in some rounds. If this is representative for other panels, it would seem that panellists who need several reminders in the earlier rounds may also need more at later stages. We would suggest making an early start when late responders need to be reminded. An advantage of sending e-mail reminders was that panellists did not need to search for their link in previous messages. Text messages and telephone reminders, on the other hand, are likely to be perceived as more personal and may therefore have more emotional impact.

We also found differences concerning the time of day when panellists submitted their responses: this was spread over 24 h of the day, most likely corresponding to their shift work as a doctor. Efficacy of the type and the timing of reminders might be influenced by this as well.

Items that resulted in consensus on non-necessity took more rounds (Fig. 4). This might be due to the fact that people generally find it harder to say ‘no’ than to say ‘yes’. In our study, panellists wanted to set a condition before saying ‘no’ to an ED item because they wanted to ensure access to proper healthcare for all patients. Taking this type of panellist behaviour into consideration prior to running a study will likely lead to a more accurate estimation of the number of rounds (resources) needed.

There is no rule that specifies which cut-off value for consensus should be used. Commonly applied levels vary between 51 and 80% [24]. It has been shown that using different consensus definitions on one data set can lead to dramatically different results [17, 25]. Interested in the effect on the results we estimate what effect increasing the cut-off value from 70 to 80% would have had on our results. We found that, in general, a majority of items showed an increase in consensus with an increasing number of rounds, although this increase became less and less steep [13]. Therefore, it seems reasonable to assume that accepted items with consensus in early rounds which varied between 70 and 80% might have resulted in higher consensus if they had been retained in another round. We can confirm this for items that were repeated in extra rounds (following our two-similar decision rule) all nine items passed the 80% mark. In Part 1, we accepted 8% (3/36) of the items within the 70–80% range, and in Part 2 this was 38% (15/63 items) and 31% (9/29) for specialist availability. Eleven of these items were accepted in a first round, four in a second round and three in a third round. In view of these findings, we conclude that changing the cut-off value would most likely not have had major effects on our results.

Response times are shown in Fig. 5 and Fig. 6. Since response times tend to be influenced by many factors, study researchers should discuss these and offer explanations or interpretations. In our first survey, it took a reminder to motivate half of the panellists to respond. We saw clear effects of sending out reminders: in the last rounds, we sent individualized reminders spread over a short period of time. This was done for logistical reasons so as to be able to contact panellists individually.

The duration of the first round in Part 2 proved to be longer than the duration of other rounds. The time needed to complete this survey was by far the longest, and therefore the research team felt that panellists should be allowed more time to submit their responses. The team did not, however, realize at that moment that response times were mostly influenced by delays in opening the survey rather than by the time that was needed to complete it. The third round was short, most likely due to the fact that by then consensus had been reached for most items. Furthermore, panellists might have had more time because the period concerned was a holiday period and reminders were sent out at an early stage. The longer duration of Round 4 was no surprise: with continuing rounds, panellists generally experience response exhaustions occurs in panellists [11], especially with busy experts and hard-pressed clinicians [10].

## Conclusions

In conclusion, this article described the methodological considerations and relevant practical aspects of our Delphi study that resulted in a 100% response rate. This exemplifies the value of a systematic approach to design choices. Based on our experience, we advise those considering a Delphi study with the aim to reach consensus on a certain topic to do the following. Adopt the CREDES guideline. Consider a two-part design, including a first part to establish a common language and to familiarize both the panellists and the research team with the online tool that is used. Invest in ensuring personal commitment from the panellists during the recruitment phase. Set clear decision rules to enhance consistency during the process and to keep the process comprehensible for the panellists. Exclude items that have reached consensus from the next rounds, but use a confirmation round in which panellist are given the option to reintroduce such items into the discussion. Design the e-Delhi in a clear and consistent lay-out throughout the full study. Expect items that result in a negative advice to require more rounds before consensus is reached. Finally, send out reminders at an early stage. Our data suggest that delays in survey responses are usually due to participants not opening a survey rather than participants taking a long time to complete it. A rigorous plan for reminders will enhance both high response rates as well as a timely completion of surveys. Adopting this overall approach may assist researchers in the future execution of Delphi studies and may help them to enhance the quality of Delphi designs in terms of improved rigor and higher response rates.

## Data Availability

The datasets used and/or analysed during the current study are available from the corresponding author on reasonable request.

## Abbreviations

CBRN: Chemical, Biological, Radiological and Nuclear
CREDES: Guidance on Conducting and REporting DElphi Studies
ED: Emergency Department
e-Delphi: electronic Delphi
EP: Emergency Physician
MRI: Magnetic Resonance Imaging
